# Intensity Distribution of Collegiate Cross-Country Competitions

**DOI:** 10.3390/sports12010018

**Published:** 2024-01-05

**Authors:** Noah Perez, Payton Miller, John W. Farrell

**Affiliations:** Clinical Biomechanics and Exercise Physiology Laboratory, Texas State University, San Marcos, TX 78666, USA; njp45@txstate.edu (N.P.); pem43@txstate.edu (P.M.)

**Keywords:** critical velocity, D′ prime, exercise, exercise intensity zones, intensity distribution, performance modeling, running

## Abstract

The primary purpose of the current investigation was to perform an intensity distribution analysis of a collegiate cross-country (CC) competition, with a secondary purpose to compare race times (RT) with modeled performance times (MPT). Participants completed an incremental treadmill test to determine gas exchange threshold (GET), while the three-minute all-out test was conducted on a 400 m outdoor track to determine critical velocity (CV) and D prime (D′). GET and CV were used as physiological markers for the intensity zones based on heart rate (HR) and running velocity (RV), while CV and D′ were used to determine modeled performance times. Participants wore a Global Positioning System (GPS) watch and heart rate (HR) monitor during competition races. Statistically, less time was spent in HR Zone 1 (12.1% ± 13.7%) compared to Zones 2 (37.6% ± 30.2%) and 3 (50.3% ± 33.7%), while a statically greater amount of time was spent in RV Zone 2 (75.0% ± 20.7%) compared to Zones 1 (8.4% ± 14.0%) and 3 (16.7% ± 19.1%). RTs (1499.5 ± 248.5 seconds (s)) were statistically slower compared to MPTs (1359.6 ± 192.7 s). The observed differences in time spent in each zone are speculated to be related to the influence of environmental conditions on internal metrics and difference in the kinetics of HR and running velocity. Differences in RTs and MPTs are likely due to the MPT equation modeling all-out performance and not considering race strategies.

## 1. Introduction

National Collegiate Athletics Association (NCAA) Cross-Country (CC) races range from 5 km to 10 km, with large variations in elevation profile between courses. Based on distance (>3 km) and duration of the race (>12 min and <2 h), CC is classified as distance running [1]. The determinants of performance during distance running have been extensively investigated and found to have significant associations with maximal oxygen uptake (VO_2_max), critical velocity (CV), running economy, and the gas exchange threshold (GET) [1,2]. To enhance physiological outcomes and improve performance, the training of distance runners is organized and periodized in a manner that allows for the accumulation of training time at specific intensities [3,4]. Researchers and sport scientists have utilized a training intensity distribution (TID) analysis in order quantify the amount of time spent in each of the exercise intensity zones and to estimate overall physiological stress accumulated during training [5,6,7].

The establishment of training intensity zones is required to perform a TID analysis and should be anchored by physiological markers that represent differences in acute responses to exercise. The GET, the convergence of VCO_2_ and VO_2_ toward a zero-difference value, and CV, the highest sustainable running velocity in which a metabolic steady state can be achieved, have been used previously to establish a three-zone model for quantifying TID for middle-to-long-distance runners, with the transition between moderate (Zone 1) and heavy exercise (Zone 2) demarcated by the GET and the transition between heavy and severe exercise (Zone 3) marked by CV [8,9,10]. The TID of both middle- and long-distance runners has been recently investigated with a systematic review indicating that a pyramidal TID is commonly observed to be followed by a polarized TID [4,11]. Although the TID of training has been investigated extensively, the intensity distribution of an actual collegiate cross-country competition has yet to be investigated. The basic principles of periodization and exercise training state that the stimuli applied during training sessions should progress in the order of non-specific to specific stimuli, with the specific stimuli phasing closely mimicking the physiological demands of the competition. However, it is unclear what the physiological demands of a collegiate cross-country competition are as this has yet to be fully investigated. Though previous investigations have reported associations between performance metrics and race times, this does not provide a clear understanding of the physiological demands of collegiate cross-country racing.

The modeling or prediction of race performance time has grown in popularity amongst coaches and sport scientists. The CV concept originated from the graphing of world-record times from various forms of human location and evolved into a method of modeling total work capacity relative to the time of reaching volitional exhaustion [12,13]. CV represents the highest velocity in which a metabolic steady state can be achieved while D′ (pronounced D prime) is the finite capacity to maintain velocities exceeding CV. An equation has been formulated using CV, D′, and the desired running distance (D) to predict the required time to reach D [13]. However, it is unclear if this performance modeling equation is applicable to a collegiate CC race, as it only predicts all-out running performance without taking consideration oscillation in running velocity or alternative race strategies [14]. Thus, the primary purpose of the current investigation is to analyze the intensity distribution of an NCAA Division 1 CC competition using both running velocity and heart rate. A secondary purpose of the current investigation was to compare actual race times with modeled performance times using CV and D′.

## 2. Materials and Methods

The current investigation utilized a nonexperimental observational study design of the racing athletes to analyze the intensity distribution of NCAA Division 1 CC competitions using both running velocity and heart rate. Male and female middle-distance runners were recruited from a NCAA Division 1 Cross-Country and Track and Field program. Participants visited the laboratory on one occasion to conduct an incremental treadmill test (ITT) to determine performance variables. Forty-eight hours after the first visit, participants completed the three-minute all-out test (3 MT) on a 400 m outdoor track to determine CV and D′. Testing was conducted the week prior to the first NCAA cross-country competition. Participants were provided with a Global Positioning System (GPS) watch (Garmin Forerunner 255, Garmin, Olathe, KS, USA) and heart rate monitor (Garmin HRM Dual, Olathe, KS, USA). Participants were instructed to wear both devices during their cross-country competitions. No manipulation of training was conducted by the research team, with training being prescribed solely by the coaching staff. All participants were informed of the benefits and risks of participating in the current investigation before providing written consent, which was obtained from all participants in accordance with the Declaration of Helsinki. This investigation was approved by the Texas State University Institutional Review Board (IRB #8353). All participants underwent the same testing procedures.

### 2.1. Participants

Ten middle-distance runners (seven males and three females) were recruited from an NCAA Division 1 CC and track and field program to participate in this investigation. Data reported are from two separate CC competition years, though they were sequential. All athletes were pre-screened for contraindications to participation in exercise training via the Physical Activity Readiness Questionnaire for Everyone (PARQ+). Additionally, participants received clearance from the team physician and coaching staff to participate in the current investigation. Participants were informed that their training would not be compromised and they would continue to follow the direction of the coaching staff.

### 2.2. Data Collection Methods

#### Incremental Treadmill Test

Participants completed a 10 min warmup on a motorized treadmill (Pro XL; Woodway; Waukesha, WI, USA) at a self-selected pace, followed by a series of warm-up drills. This warm-up reflects the athletes’ normal warm-up protocol completed prior to training sessions. The incremental treadmill test (ITT) consisted of 1 min stages with a set incline of 1% with speed increasing by 0.8 km·h^−^^1^ every minute until volitional fatigue was met. Initial speed was set at 12 km·h^−^^1^ and 11.2 km·h^−^^1^ for males and females, respectively [3]. Heart rate was assessed via chest strap heart rate monitor (HRM Dual, Garmin, Olathe, KS, USA) while inspired and expired gases were collected (7450 Series Silicone V2 Oro-Nasal Mask, Hans Rudolph, Shawnee, KS, USA) and analyzed via an open circuit spirometry system (TrueOne 2400, Parvo Medics, Sandy, UT, USA). Oxygen consumption (VO_2_), maximal oxygen consumption (VO_2_max), carbon dioxide production (VCO_2_), and minute ventilation (V_E_) were determined from inspired and expired gases. VO_2_max was defined as the highest 20 s average for oxygen consumption. GET, along with the corresponding velocity and heart rate, was determined following the procedures recommended by Beaver et al., with a time-delay of 1 min used for interpolation of speed-evoking GET. Briefly, GET is described as a convergence of VCO_2_ and VO_2_ toward a zero-difference value and is detected by examining an inflection in a VO_2_ using a VCO_2_ plot [2,15].

## 3. MT

The 3MT was conducted 48 h after the ITT on an outdoor 400 m running track during minimal wind and ambient temperature conditions (wind: <2.5 m/s’ temperature: 19–25 °C; relative humidity: 30 to 40%. Prior to the 3MT, participants were assigned a heart rate monitor and GPS watch to collect heart rate and velocity data at 1 Hz during the assessment. Participants completed a 10 min warmup at a self-selected pace on the running track before completing a series of warmup drills. Participants then rested for 5 min before beginning the 3MT. The test was initiated from a stationary position, and participants were instructed to run as fast as possible for three minutes and five seconds. The additional five seconds follow the recommendation to account for time to reach maximal velocity [13,16]. Participants were blinded to elapsed time and were provided strong verbal encouragement throughout. Using the derived velocity–time curve, CV and D′ were determined as the velocity over the final 30 s of the assessment and the area under the curve above CV, respectively. The heart rate associated with CV was determined using the heart rate and velocity relationship during the ITT.

### 3.1. Exercise Intensity Zone Determination

Three exercise intensity zones were established for both velocity and heart rate using variables determined from the ITT and 3MT. Moderate intensity, or Zone 1, was established as heart rate and velocity values < GET; heavy intensity, or Zone 2, was established as between GET and CV; and severe intensity, or Zone 3, was established as >CV. Heart rate monitors and GPS watches were worn during NCAA Division 1 cross-country competitions with distances ranging from 5 km to 8 km. Data from each competition was downloaded to a CSV file and the total time spent within each of these zones for both heart rate and velocity were tabulated.

### 3.2. Performance Modeling

Using the following equation, the theoretical performance for runners at each race distance completed was determined with D equaling the desired running distance in meters, D′ equaling D prime expressed in meters, and CS equaling critical speed expressed in meters per seconds [13].

$$\frac{D-D^{\prime }}{CS}$$

### 3.3. Statistical Analysis

Results are presented as mean ± standard deviation unless otherwise stated. Descriptive statistics were used for demographic data. Two-way analysis of variance (ANOVA) was used to determine if statistically significant interactions were present between sex and time in zone using both running velocity and heart rate with Bonferroni pairwise comparisons used to identify significant differences. If no significant differences between sex were present for both running velocity and heart rate, then groups would be collapsed and a two-way analysis of variance (ANOVA) would be used to determine if statistically significant interactions were present between time in zone and method of calculation (running velocity vs. heart rate) with Bonferroni pairwise comparisons used to identify significant differences. Pair samples *t*-test were used to compare values of modeled performance time and actual race time. Cohen’s effect size (*d*) was used to assess the magnitude of differences with <0.2, 0.2 to 0.6, >0.6 to 1.2, >1.2 to 2.0, and >2.0 to 4.0 classified as trivial, small, moderate, large, and very large, respectively [17]. Pearson’s partial correlation coefficient was used to determine the relationship between modeled performance time and actual performance time while controlling for VO_2_max. If a statistically significant association was present, regression analysis was conducted to determine the standard error of the estimate (SEE) in addition to 95% limits of agreement (LOA). Correlation coefficient values of ≤0.3, >0.3 and ≤0.5, and >0.5 were interpreted as small, moderate, and large, respectively [18]. Statistical significance was set at *p* ≤ 0.05. Statistical analysis was completed with SPSS v.26 (IBM Corp., Armonk, NY, USA).

## 4. Results

A total of 24 race files were analyzed from the 10 participating athletes—16 from males and 8 from females. Participant demographic information and performance parameters are reported in Table 1. In total, four courses were run during the season, with course profile information reported in Table 2.

**Figure 1:**
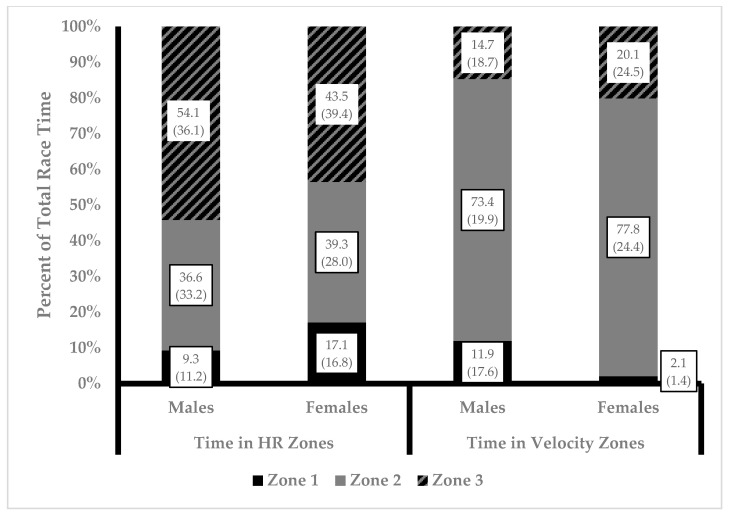
Percent of race time spent in intensity zones for males and females. Twenty-four total race files were analyzed (16 from 7 males and 8 from 3 females) and included in the analysis. Data presented as mean (standard deviation).

**Figure 2:**
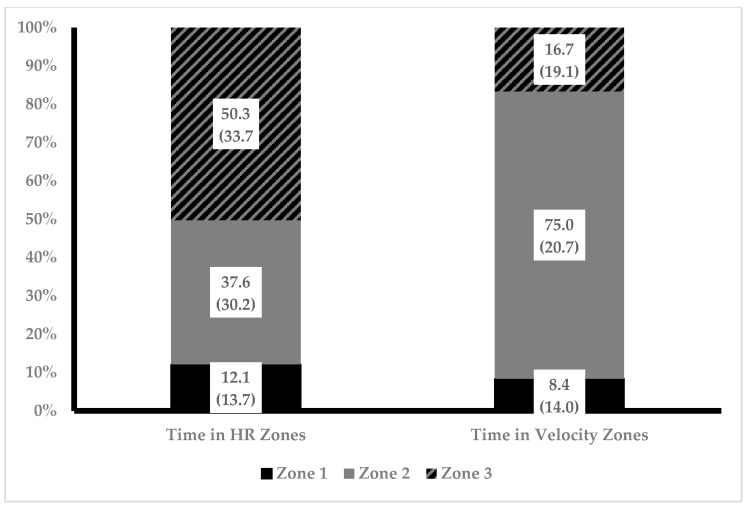
Percent of race time spent in intensity zones for males and females. Twenty-four total race files were analyzed (16 from 7 males and 8 from 3 females) and included in the analysis. Data presented as mean (standard deviation).

The percentage of competition time spent in each zone based on heart rate and running velocity separated by sex is illustrated in Figure 1. Both running velocity and heart rate data, not the distribution between zones, were observed to not violate sphericity. Two-way ANOVA revealed no statistically significant interactions between sex and percent of race time spent in zones were present. A main effect was present for percent of race time in zones for both running velocity (F = 82.8, *p* = 0.00, η^2^ = 0.734) and heart rate (F = 8.823, *p* = 0.000, η^2^ = 0.227). Post hoc analysis revealed a statistically significant difference between percentage of time spent in running velocity Zone 2 compared to Zone 1 (75.0% ± 20.7 vs. 8.4% ± 14.0, *p* = 0.00, d = 3.7) and Zone 3 (75.0% ± 20.7 vs. 16.7% ± 19.1, *p* = 0.00, d = 2.9). Post hoc analysis revealed a statistically significant difference between percent of race time spent in heart rate Zone 1 compared to Zone 2 (12.1% ± 13.7 vs. 37.5% ± 30.2, *p* = 0.01, d = 1.0) and Zone 3 (12.1% ± 13.7 vs. 50.3% ± 33.7, *p* = 0.00, d = 1.4). Groups were collapsed across sex, and percentage of race time spent in zones was compared between heart rate and running velocity. Time in zone for both methods is illustrated in Figure 2. Two-way ANOVA revealed no statistically significant interactions between method of calculation and percent of race time spent in zones were present. However, a main effect for zone was present (F = 43.1, *p* = 0.00, η^2^ = 0.407). Post hoc analysis revealed a statistically significant difference percent time spent in Zone 1 compared to Zone 2 (10.2% ± 13.8 vs. 56.2% ± 31.8, *p* = 0.00, d = 1.8) and Zone 3 (10.2% ± 13.8 vs. 33.3% ± 32.9, *p* = 0.00, d = 0.9) and between Zone 2 and Zone 3 (56.2% ± 31.8 vs. 33.3% ± 32.9, *p* = 0.00, d = 0.7).

**Figure 3:**
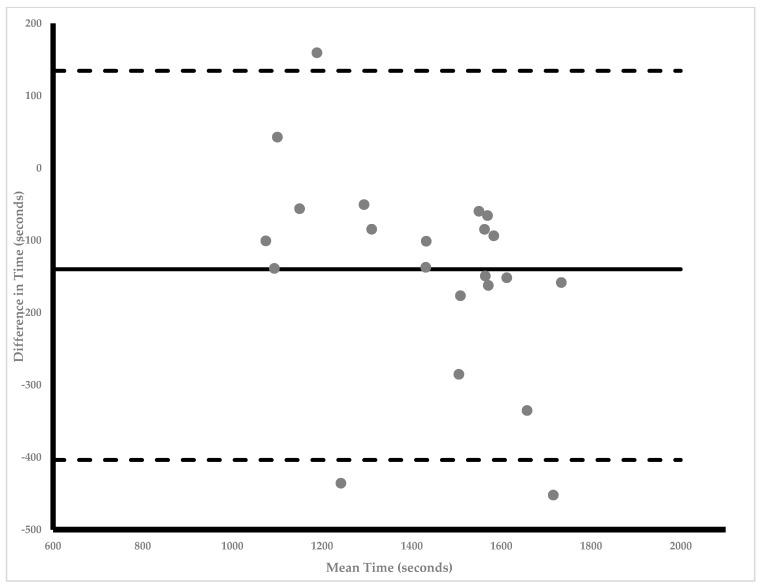
Upper and lower 95% confidence intervals of modeled performance times. Twenty-four total race files were analyzed (16 from 7 males and 8 from 3 females) and included in the analysis.

Athletes’ average velocities over the duration of competitions were 96.2% ± 11.3 of their CV. Paired samples *t*-tests revealed a statistically significant difference with a moderate effect size between modeled performance time and actual race time (1359.6 ± 192.7 s vs. 1499.5 ± 248.5 s, *p* = 0.00, d = 0.62). However, partial correlation analysis revealed a large statistically significant association between modeled performance time and actual race time (r = 0.683, *p* = 0.00) when controlling for VO_2_max. Figure 3 illustrates a mean difference of −139.9 ± 139.3 s (95% LOA: −403.5 to 134.5) was observed. A statistically significant regression (r^2^ = 0.688, *p* = 0.168) with an SEE of 142.1 s was observed.

## 5. Discussion

The primary purpose of the current investigation was to analyze the intensity distribution of an NCAA Division 1 CC competition by quantifying the percentage of time spent in each of the three exercise intensity zones using both running velocity and HR. To the knowledge of the current authors, this has yet to be reported. It was observed that athletes spent a statistically higher percentage of race time in Zone 2 compared to Zones 1 and 3 based on running velocity and a statistically higher percentage of time spent in Zones 2 and 3 compared to Zone 1 based on HR. No statistically significant differences were observed between sexes for time spent in each of the zones, nor were there any differences based on the method of quantification. Athletes maintained an average running velocity of ~96% of their CV across competitions. Additionally, a secondary purpose of the current investigation was to compare actual race times with modeled performance times using CV and D′. A mean difference of 139 s was observed between modeled performance time and actual race time. Understanding the intensity distribution of a collegiate CC competition and the ability to model and predict performance will help inform coaches and athletes in their race preparation and strategy.

Collegiate CC running places a significant amount of physiological and metabolic stress on athletes, requiring them to race for an extended duration, across multiple exercise intensity zones. An understanding of physiological mechanisms that take place within each zone is the foundation of understanding CC racing’s physiological demands. Zones 1, 2, and 3 are noted as the moderate, heavy, and severe exercise intensity domains, respectively. The boundary between Zone 1 Zone 2 is demarcated by the gas exchange threshold, while transition from Zone 2 to Zone 3 is demarcated by CV [2]. Exercise within each zone induces unique acute physiological responses [8]. These responses are primarily associated with the magnitude of perturbation from homeostasis and the ability or inability to achieve a metabolic steady state, which is defined as a leveling out or plateauing of parameters such as blood lactate and VO_2_ during constant-work-rate exercise [19,20,21,22,23]. Zone 1 is characterized by sustained work that is performed below the gas exchange, ventilatory, or lactate threshold with an increase in VO_2_, with a metabolic steady state achieved in ~3 min from the onset of exercise. Exercising within Zone 2 is characterized by an elevated VO_2_ and blood lactate response, coinciding with the development of the VO_2_ slow component, though a metabolic steady state is still achievable within 10–20 min from the onset of exercise [2,24]. Increases in both central and peripheral nervous system fatigue with reductions in peak power output, maximal voluntary contraction, and VO_2_max have all been observed following prolonged exercise within Zone 2 [25]. The transition from Zone 2 to Zone 3 is demarcated by CV, representing the highest exercise intensity in which a metabolic steady state can be achieved [8,23,26,27]. Thus, exercise within Zone 3 is primarily characterized by the failure to achieve a metabolic steady state, with both VO_2_ and blood lactate increasing significantly from the onset of exercise and continuously rising at a rate proportional to the distance above CV until achieving VO_2_max and task failure [22,28,29].

The current investigation observed statistically significant differences in the time spent in each of the zones based on velocity, with ~8%, ~75%, and ~16% of race time spent in Zones 1, 2, and 3, respectively. The distribution of percentages of time for velocity zones reflects the athletes self-selecting at a high velocity that is near their CV but still below, allowing athletes to still reach a metabolic steady state and avoiding the rapid accumulation of fatigue associated with exercising in Zone 3 continuously [21,30]. However, ~16% of race time was spent within Zone 3, conveying that the athletes spent time running above their CV, thus utilizing D′. Based on the time spent within each zone, it appears that athletes were strategic in their decision to push their velocity into Zone 3 and use the finite D′ in instances such as passing an opponent, matching increases in opponents’ velocities, or adjusting to alterations in course elevation. Previous investigations on self-selected pacing during time-trials in experienced runners have observed a well-defined u-shaped pattern, beginning at intensities within Zone 3 and then decreasing to a sustainable intensity that falls within Zone 2 for much of the exercise duration before increasing back into Zone 3 towards the end of the time-trial [9,31]. The decrease in intensity from within Zone 3 to Zone 2 not only aligns with maintaining a desirable intensity but also allowing for the regeneration of D′ in a manner proportional to the distance below CV. The replenishment of D′ may allow athletes to perform the “kick” at the end of the time trial or race. In the context of racing, rather than solo time-trial performance, an athlete may accelerate and decelerate their velocity above and below CV multiple times depending on the tactics of the race [14].

For HR, a statistically greater amount of time was spent in Zones 2 (~38%) and 3 (~50%) compared to Zone 1 (~12%). Although no statistically significant differences were different based on method of quantification, this contrasts with the distribution of intensity based on velocity, as the majority of time was spent in Zone 2 (~75%), whereas majority of time was spent in Zone 3 based on HR. It is speculated that cardiovascular drift (CD) is likely the reason there is a substantial variance between corresponding heart rate and velocity. Cardiovascular drift is characterized as the slow and steady increase in heart rate that is seen during extended bouts of endurance exercise despite a similar workload and intensity. It is hypothesized to occur due to fluid loss to perspiration—thus, the total volume of blood circulating decreases, leading to decreases in stroke volume. In order to maintain the same cardiac output despite decreases in stroke volume, the heart increases the rate of contraction in order to maintain circulation and delivery of oxygen to working muscles [32]. An additional hypothesis suggests that due to increases in skin temperature, blood will begin to pool at the surface of the skin to promote the dissipation of heat through convection [32]. This divergence in blood flow also leads to decreases in total blood volume returning to the heart, resulting in the heart having to increase the rate of contraction to counteract decreases in stroke volume to maintain cardiac output. Although the development of cardiovascular drift assists individuals in maintaining a constant work rate despite increases in core and skin temperature, perceived exertion has been observed to increase when cardiovascular drift manifests [31]. Dehydration has been reported to contribute to the development of cardiovascular drift, but hydration and nutritional status were not assessed prior to races in the current investigation.

An interesting observation of the current investigation is the lack of statistically significant differences overserved between the distribution of race time when quantified using running velocity and HR. The authors speculate that this is likely due to the large variances observed within zones using the two different methods. The large variances may be attributed to differences in environmental conditions on competition days, racecourse profiles, and differences in the kinetics of internal and external metrics of intensity. This is likely due to some of the physiological responses to hot and humid atmospheric conditions that can influence HR but also due to some of the inherent differences in internal versus external measures of intensity. External measures represent the physical work performed during training sessions and competitions (i.e., running velocity), while internal measures encompass the corresponding physiological response (i.e., heart rate) to the performed work [33]. A previous investigation of the TID of middle-distance runners over an 8-week period observed differences in TID when quantified based on running velocity and HR [3]. A greater amount of time was spent in Zone 2 when using heart rate compared to running velocity. It was speculated that this was due to slower kinetics of heart rate in response to changes in running velocity (e.g., running velocity would increase very quickly but HR took longer to respond to the change in velocity). Although less time was spent in Zone 2 and more time in Zone 3 for HR compared to running velocity, the same difference in response time between velocity and HR may contribute to the large variances observed during competition. Athletes will accelerate, decelerate, and cross over between Zones 2 and 3 very quickly in terms of running velocity, but due to the slower HR kinetics, HR may still stay elevated even though velocity has decreased and may subsequently slowly decrease. HR will also be influenced by the atmospheric conditions that day, potentially resulting in a disassociation between alterations in velocity and alterations in HR. Additionally, an acute dose–response relationship exists between these two modes of measures and is in constant flux due to different states of fatigue. For example, a running velocity of 5.0 m/s may elicit a corresponding heart rate of 175 bpm during a training session after coming off a day of recovery. However, after several days of training including extended bouts of running in Zone 3, the same 5.0 m/s may elicit an HR of 185 bpm due to accumulated fatigue and altering the distribution of intensity between zones [14]. The current investigation did not assess fatigue levels prior to competition and did not have access to training logs leading up to competitions.

A major benefit of assessing CV and D′ is the ability model total work capacity which can then be used to inform running race strategy and to prescribe high intensity intermittent training [25,30]. However, the modeled race time was statistically lower than actual race time with a large SEE and large LOA observed. This is likely due to the equation not accounting for oscillations in running velocity that are dictated by the competition, strategy, and terrain of the racecourse. As previously mentioned, a common pacing strategy is to begin in Zone 3 before decreasing running velocity to below CV in Zone 2, for much of the exercise duration before increasing again into Zone 3 near the end of race [31]. The equation utilized models the performance time as if the individual ran to their physiological limit on a flat surface, such as an outdoor running track. This likely does not reflect what is done by most individuals in races, in which the U-shaped pacing strategy is most likely implemented and does not reflect the characteristics of racecourses for collegiate CC [7]. It should be noted that participants and coaches were not made aware of their CV, D′, or modeled performance time and thus did not have the ability to use this in the development of race strategy. Future investigations should seek to better understand how these variables could be implemented by coaches and athletes in actual race scenarios.

The current investigation is not without limitations. A limited number of collegiate cross-country runners were able to be recruited for the current investigation, with more males than females participating, limiting the generalization of the results. Training logs were not available for analysis, making it unclear the degree of accumulated fatigue each participant had going into each of the competitions. Additionally, all performance metrics were assessed at the beginning of the competition phase of training. Though traditionally, this phase of training primarily targets the maintenance of physiological adaptations induced during previous phases of training, participants may have improved metrics—such as the velocity associated with GET and CV—thus altering zones.

## 6. Conclusions

In conclusion, the current investigation reports that the majority of race competition time is spent in Zone 2 when quantified based on running velocity, but when quantified based on HR, the majority of time is spent in Zone 3. The observed differences, though not statistically different based on quantification of internal or external measures of intensity, have been reported previously in middle-distance runners and are thought to be related to the influence of environmental conditions on internal metrics and difference in the kinetics of HR and running velocity [33]. Additionally, the modeled performance times using CV and D′ were statistically lower (i.e., faster) when compared to actual race times. This is likely due to the equation not accounting for oscillations in running velocity, the implementation of different pacing strategies, and changes in terrain the influence running velocity. To the current authors knowledge, this is the first investigation to quantify the demands of a collegiate CC race using both internal and external metrics of intensity. The data provided in the current investigation can inform athletes and coaches on where pacing falls in relation to performance metrics, such as CV and GET, which can then be used to inform training.

## Figures and Tables

**Table 1:** Participant demographic information and performance parameters (n = 10).

	Males (n = 7)		Females (n = 3)	
Variable	Mean ± SD	Range	Mean ± SD	Range
**Age (y)**	19 ± 2	18–23	20 ± 1	19–21
**Height (cm)**	180 ± 5	174–188	165 ± 3	162–167
**Body Mass (kg)**	70 ± 6	60–80	57 ± 2	56–60
**VO_2_max (mL/kg/min)**	64.5 ± 6.9	53.6–75.0	50.4 ± 0.8	49.7–51.4
**CV (m/s)**	5.3 ± 0.3	4.7–5.8	4.6 ± 0.3	4.3–4.8
**D′ (m)**	108.6 ± 48.0	55.5–206.3	77.4 ± 59.7	26.0–142.9
**HR at CV (bpm)**	187 ± 6	176–194	189 ± 14	173–199
**GET (m/s)**	4.2 ± 0.2	3.9–4.6	3.7 ± 0.2	3.5–3.9
**HR at GET (bpm)**	165 ± 7	158–177	171 ± 8	163–179
**Race time (s)**	1657 ± 126	1483–1942	1223 ± 137	1079–1460

VO_2_max = maximal oxygen consumption; CV = critical velocity; D′ = d prime; HR = heart rate; GET = gas exchange threshold.

**Table 2:** Course profile information taken from global positioning system watch data.

Course	Sex	Number of Race Files	Distance (km)	Elevation Gain (m)	Elevation Loss (m)
1	Male	6	7.9	46.0	42.1
	Female	2	5.9	35.1	36.0
2	Male	3	8.0	14.0	14.0
	Female	3	5.0	26.0	25.0
3	Male	5	8.0	43.9	46.0
	Female	2	6.0	29.9	31.1
4	Male	2	7.8	49.1	45.1
	Female	1	5.1	32.9	32.9

km = kilometer and m = meters.

## Data Availability

The data presented in this study are available upon request from the corresponding author.
